# Functional and Clinical Outcomes of Total Knee Arthroplasty: A Prospective Study

**DOI:** 10.7759/cureus.52415

**Published:** 2024-01-16

**Authors:** Chintan Chaudhary, Udit Kothari, Samarth Shah, Deval Pancholi

**Affiliations:** 1 Department of Orthopedics, Gujarat Medical Education and Research Society (GMERS) Medical College, Gandhinagar, IND; 2 Department of Orthopedics, Aashray Orthopaedic Hospital, Modasa, IND; 3 Department of Orthopedics, Gujarat Medical Education and Research Society (GMERS) Medical College, Vadnagar, IND; 4 Department of Orthopedics, Smt. Nathiba Hargovandas Lakhmichand (NHL) Municipal Medical College, Ahmedabad, IND

**Keywords:** mechanical angle, kss, patella, mechanical axis, tka

## Abstract

Introduction: A key to successful patient outcomes following knee procedures like total knee arthroplasty (TKA) is achieving normal knee alignment. Normal mechanical balance in the joints must be restored in order to lessen the failure rate of TKA, and precise assessment of lower extremity alignment and component location is thought to be the primary determinant of a good clinical outcome over the long term.

Objective: The aim of this study was to assess the early functional and clinical outcomes of TKA by analyzing post-operative mechanical axis and Knee Society Score (KSS) data. The study also aimed to investigate the importance of achieving neutral mechanical alignment in TKA for long-term results.

Methods: A prospective and retrospective observational study was conducted, involving 40 patients with primary osteoarthritis who underwent bilateral or unilateral TKA. Demographic information, pre-operative comorbidities, knee abnormalities, and radiographic assessment were collected. Post-operative mechanical axis and implant placement were evaluated using CT scanograms. Clinical and functional assessments were performed using the Knee Society Score at regular intervals. The KSS scores were obtained at six-month intervals following surgery. The scores were categorized as excellent, very good, good, fair, or poor based on predefined criteria.

Results: A total of 47 knees from 40 patients were analyzed. The mean age of the study population was 65.6 years. Female patients accounted for 67.5% of the sample. The majority of patients (57.4%) achieved an excellent KSS score, followed by very good (25.5%) and good (17%) scores. The mean KSS score significantly improved from 177 points pre-surgery to 225 points post-surgery. The post-operative mechanical axis ranged from 1.1 degrees valgus to 9 degrees valgus, with a mean value of 3.5 degrees valgus. The range of motion improved from 10 to 90 degrees post-operatively, with a mean range of 0-110 degrees.

Conclusion: This study suggests that total knee replacement surgery resulted in positive outcomes, with most patients achieving excellent or very good scores on the Knee Society scale. Proper axial and rotational alignment of the components during TKA enhanced functional outcomes and contributed to long-term clinical success. Restoration of neutral mechanical alignment and optimal component placement may lead to lower revision rates and increased durability after TKA. Further studies with larger sample sizes are needed to validate these findings.

## Introduction

India, a nation of 1.2 billion people, has a sizable population with knee arthritis. Osteoarthritis of the knee is the most prevalent articular disease in the world and a main source of chronic impairment, particularly in the senior population [1, 2]. There is a discrepancy between the anatomical status of the joint on imaging or clinical examination on the one hand, and symptoms and disability on the other, due to the heterogeneous disease process that affects several joints [3].

Since the 19th century, the idea of enhancing knee joint function by altering the articular surfaces has drawn interest. From soft tissue interposition arthroplasty through resection arthroplasty to surface replacement arthroplasty, the surgical approaches have diversified. Several types of prostheses were created for surface replacement arthroplasty to address the complex knee kinematics [4].

The most popular and effective surgical treatment for end-stage osteoarthritis is total knee arthroplasty (TKA), which provides patients with pain relief, functional rehabilitation, and a better quality of life. It is a bony and primarily soft-tissue treatment where the coronal plane and component alignment have received a lot of attention [5]. The knee's neutral alignment, which lessens mechanical and shear loads on the bearing surfaces and the bone-prosthesis interface, is largely responsible for the long-term success of TKA. Moreover, neutral alignment assists in harmonizing the pressures passed through the knee joint's soft tissue sheath [6].

TKA depends on a lot of variables for it to be successful in terms of pain alleviation, increased function, higher patient satisfaction, and implant longevity [7]. These factors include those related to the prosthesis, such as implant size, tribology, geometry, alignment, and position; those related to the patient, such as size, weight, activity level, the presence of medical comorbidities, psychological and physiological reaction to joint surgery; and those related to the surgery itself, such as surgical skill and experience, length of procedure, appropriate pre-operative planning, and prosthesis implantation. The link between surgical technique and implant performance and survival has received more attention recently [8].

The geometry of the component parts, the degree of conformance of the articulating surface, and the anchoring method are all features that can be found in different systems [9]. The decision to align follows the long-held principle that the lower limb's post-operative alignment should be within ±3° of the neutral mechanical axis [10]. One of the essential requirements for a successful TKA is the restoration of this neutral mechanical alignment of the knee. It is believed that proper knee alignment will lessen the mechanical and shear pressures exerted on the bearing surfaces as well as the bone/prosthesis interfaces following TKA, making it one of the most important elements in determining long-term results. Furthermore, good alignment helps to balance the forces that are transferred through the soft-tissue envelope, which is essential for the joint to operate properly [11].

Total knee arthroplasties that are misaligned might reduce implant survival rates, as well as increase wear, produce less-than-ideal functional results, and fail early, causing component loosening with older polyethylene and implant designs. To achieve this purpose, a variety of surgical procedures and alignment tactics have been used. TKA has frequently employed either the measured resection or gap balancing methods of classical alignment to reduce surgical trauma through less invasive surgery and better implant positioning [12]. Patterns of femorotibial tracking are connected to shifts in the mechanical axis away from neutral, which can cause aberrant loads at the bearing surface and hasten wear [13]. The creation of various technologies, such as computer navigation and patient-specific instrumentation, aims to aid the surgeon in more accurately simulating the knee's neutral mechanical axis. Although these advancements have occasionally resulted in better radiographic alignment and fewer axis outliers, they haven't always improved clinical results. With the introduction of these many forms of prosthesis, it was important to undertake research to evaluate the effectiveness of various prostheses because the results that have been reported so far are still ambiguous. As a result, the study's goal is to assess the early functional and clinical results of total knee arthroplasty using post-operative mechanical axis and Knee Society Score (KSS) data.

## Materials and methods

Participants

The study protocol was reviewed and approved by the Institutional Ethics Committee of HM Patel Center for Medical Care and Education, Anand, Gujarat (IEC/HMPCMCE/122/FACULTY/2/189/90). A total of 40 patients with primary osteoarthritis who received bilateral or unilateral total knee replacement and gave written informed consent at the Shree Krishna Hospital, Ahmedabad, Gujarat between February 2020 and March 2021 were the subjects of a prospective and retrospective observational study. Patients who underwent total knee replacement with a fracture of the ipsilateral or contralateral limb were excluded from the study as well as those with active infection of the knee or anywhere in the body, secondary osteoarthritis-post traumatic/post-inflammatory/post-infection, and rheumatoid arthritis (RA) involving other joints of the body. All of the patients had a thorough medical history, demographic information, pre-operative medical comorbidities, pre-operative knee abnormalities from case studies, radiographic assessment of arthritis using x-rays, and pre-operative mechanical axis using a CT scanogram.

Surgery

After a comprehensive pre-op evaluation, the surgeon's team took each patient up for surgery. The patient was placed in the supine position with their knee flexed to 90 degrees. All patients were given tranexamic acid (15 mg/kg, slow IV infusion) 10 minutes prior to deflation of the tourniquet. All patients underwent general anesthesia consisting of propofol induction, vecuronium bromide to facilitate intubation, and oxygen/air/isoflurane for maintenance of anesthesia. Using a tourniquet, sterile preparation was carried out from the thighs to the toes and the area was wrapped. The majority of the knees were operated on utilizing the MAXX FREEDOM posterior cruciate replacement TKA prosthesis; however, all the knees were operated on. Knee system, a few patients underwent surgery using the STRYKER knee system, while others underwent surgery using the PFC SIGMA (J&J) system.

An anterior midline retinacular incision of 3 to 5 cm above the superior pole of the patella and extended distally to below the level of the tibial tubercle was made with the knee in 90-degree flexion. The deteriorated femoral condyle was revealed, the patella was retracted laterally and everted, and a Homan retractor was used to elevate the subperiosteal soft tissue sleeve. In cases of varus knees, the periosteum was further stripped medially from the tibia before the semimembranosus muscle insertion was liberated from the posteromedial tibia and continued distally on the anteromedial aspect of the tibia.

The crosshead with a pin, resection guide, and ankle yoke made up the extramedullary tibial guide. The resection guide was aligned at the ankle using the adjustment screw. The alignment rod was positioned parallel to the center of the tibial tuberosity to a point medial to the midpoint of the ankle joint, and the long axis of the tibial resection guide was made parallel to the tibia so that the anterior notch in the tibial tray matched the center of the tibial tuberosity. A few millimeters below the lowest articular surface, there was a resection slot (usually medial). An osteotome was used to make the last tibial cut in order to avoid excessive posterior saw blade penetration that could have cut the popliteal artery.

After exposing the femoral condyles, the Whiteside line and the trans-epicondylar line were drawn across them, and a starting hole was produced at their intersection. The distal femur was removed using the +4 mm resection slot, which allowed for a 13 mm removal from the protruding distal condyle, or the conventional resection slot, which allowed for a 9 mm removal. In order to achieve a distal cut that is perpendicular to the mechanical axis, the distal resection and valgus alignment guides were assembled onto the intramedullary alignment rod with a 5 to 7-degree valgus cut. The flexion gap and the extension gap, both with a minimum base of 10 mm, were maintained.

The A-P femoral sizer was set up so that the stylus and feet of the sizer made contact with the femur's shaft and the feet with the posterior condyles of the resected distal femur. On the medial and lateral sides of the block, 3.2 mm diameter pointed pins were used to support it against the bone. The trial tibial base, which is the same size as the femoral implant, was put together and pressed up against the proximal tibial surface. An entry hole was created for the tibial stem using the 1/2" drill guide and large reamer. An alignment rod can be inserted through the handle to check alignment to the ankle.

The guide allowed the punch to slip through. Until the punch is completely seated, use the threaded punch handle and the corresponding keel punch. The trial tibial insert was inserted onto the trial base while the knee was flexed, and an appropriate size femoral trial was placed on the distal femur using the femoral impactor. In order to prepare the cut surfaces of the femur and tibia for the implantation of the femoral and tibial components, bone cement was applied to the surfaces. The suitable tibial insert was then locked into place.

Post-operative procedure

Enoxaparin (40 mg/4,000 U) was administered subcutaneously to all patients with normal renal function 12 hours after surgery as thromboprophylaxis. The patients were put on antibacterial and analgesic IV medicines and instructed to remain strictly supine for the first day. Depending on the patient's tolerance for pain, mobilization began the evening before the operation. The sixth post-operative day saw the continuation of antibiotic treatment. After 48 hours following the operation, the suction drain was withdrawn.

Outcome evaluation

One month after surgery, the patients were examined for any indications of a post-operative infection, and x-rays were also taken. Patients were evaluated clinically and functionally using the KSS at intervals of 6 months following surgery once early post-operative problems had been ruled out. Repeat x-rays and CT scanograms were also performed to evaluate the mechanical axis and implant placement, respectively.

## Results

This study involved the analysis of 47 knees from 40 patients who had undergone total knee replacement surgery at the Department of Orthopedics in Shree Krishna Hospital between January 2020 and March 2021. Table 1 outlines the patients' demographic characteristics. The average age of the study population was 65.6 years, with the youngest patient being 47 years old and the oldest being 87 years old. The most commonly operated age group was 61-70 years (20 knees), while only one knee each was operated on in the 40-50 years and 81-90 years age groups. In the present study female (67.5%) have a higher incidence of osteoarthritis knee as compared to male (32.5%) patients.

**Table 1:** Demographic characteristics of patients

Age	Number of patients (%)
41-50	1 (2.5)
51-60	10 (25)
61-70	20 (50)
71-80	8 (20)
81-90	1 (2.5)
Gender	
Male	13 (32.5)
Female	27 (67.5)

The total knee replacement was performed on 16 (42.5%) patients on the left side and 17 (40%) patients on the right side as shown in Table 2. A total of 7 (17.5%) patients had undergone bilateral knee replacement. Patellar replacement surgery was carried out in just 5 out of the 47 knees included in the study, which represents a relatively low proportion of only 10.6%.

**Table 2:** Fracture characteristics and patella replacement

Side	Frequency (%)
Left	16 (42.5)
Right	17 (40)
Bilateral	7 (17.5)
Total	47 (100)
Patella replacement	
Done	5 (10.6)
Not done	42 (89.4)

Table 3 presents the distribution of knee society scores, categorized by grading. The majority of patients (57.4%) received an excellent score, followed by 25.5% of patients with a very good score, and 17% of patients with a good score. The total number of patients included in this analysis was 47. These results suggest that knee replacement surgery is associated with a high rate of positive outcomes, with most patients achieving an excellent or very good score on the knee society scale.

**Table 3:** Grading of Knee Society Score

Grade	Frequency (%)
Excellent	27 (57.4)
Very good	12 (25.5)
Good	8 (17)
Total	47 (100)

Table 4 presents a comparison between KSS, mechanical axis, and range of motion before and after knee replacement surgery. The mean KSS improved significantly from 177 points pre-surgery to 225 points post-surgery, with a mean score of 208.4. The mechanical axis measurement before surgery ranged from 5 degrees valgus to 9.63 degrees varus, while after surgery, the range was 1.1 degrees valgus to 9 degrees valgus, with a mean value of 3.5 degrees valgus. The pre-operative range of motion varied from 30 to 90 degrees, while post-operatively, the range was between 10 to 90 degrees, with a mean range of motion of 0-110 degrees.

**Table 4:** Comparison between Knee Society Score, mechanical axis, and range of motion after surgery

	Minimum	Maximum	Mean
Knee Society Score (Out of 230 points)	177	225	208.4
Mechanical axis (pre-operative) (degree)	5 (valgus)	27 (varus)	9.63 (varus)
Mechanical axis (post-operative) (degree)	1.1 (valgus)	9 (valgus)	3.5 (valgus)
Pre-operative range of motion (degree)	30-90	0-100	10-90
Post-operative range of motion (degree)	10-90	0-120	0-110

Table 5 presents a comparison between knee society score (KSS) and post-operative mechanical angle in two groups, where Group A had a post-operative mechanical angle less than or equal to 3 degrees and Group B had a post-operative mechanical angle greater than 3 degrees. Within Group A, 53.2% of patients had an excellent KSS score, while 46.8% of patients had an excellent KSS score in Group B. In Group A, 56% of patients had a post-operative mechanical angle less than or equal to 3 degrees, while 59.1% of patients in Group B had a post-operative mechanical angle greater than 3 degrees. The total number of patients included in this analysis was 47, with a distribution of 8, 12, and 27 patients in the Good, Very Good, and Excellent KSS categories, respectively.

**Table 5:** Comparison between Knee Society Score and post-operative mechanical angle

			Knee Society Score (KSS) category			Total
			Good	Very good	Excellent	
Post-operative mechanical axis	Less than equal to 3 (<=3) GROUP A	Frequency (Number of patients)	4	7	14	25
		% within angle_post	16.0%	28.0%	56.0%	100.0%
		% within KSS_category	50.0%	58.3%	51.9%	53.2%
	Greater than 3 (>3) GROUP B	Frequency (Number of patients)	4	5	13	22
		% within angle_post	18.2%	22.7%	59.1%	100.0%
		% within KSS_category	50.0%	41.7%	48.1%	46.8%
Total		Frequency (Number of patients)	8	12	27	47
		% within angle_post	17.0%	25.5%	57.4%	100.0%
		% within KSS_category	100.0%	100.0%	100.0%	100.0%

## Discussion

The two alignment parameters in the lower extremities are the mechanical axis and the anatomical axis. One of the most crucial elements affecting surgical prognosis is the mechanical axis, which is the axis or line of weight bearing through the bone [14]. For successful patient outcomes following knee procedures like TKA, achieving normal knee alignment is essential. The mechanical axis of the femur differs from the anatomical axis; the former is 5-7 degrees valgus to the latter [15]. The loading of the articular surfaces of the knee is greatly impacted by valgus or varus malalignment. Animal models have demonstrated that this misalignment accelerates the advancement of osteoarthrosis in the knee [16,17].

Adult patients with inflammatory arthritis, tibial malunion, physeal arrest, or tibial plateau fracture are more likely to experience valgus deformity [18, 19]. Bone components include metaphyseal femur and tibial plateau remodeling, lateral cartilage erosion, and lateral condylar hypoplasia. Soft tissue variables that contribute to the valgus deformity include the tightening of lateral tissues, such as the lateral head of the gastrocnemius, posterolateral capsule, popliteus tendon, hamstring tendons, and iliotibial band [20].

Normal mechanical balance in the joints must be restored in order to lessen the failure rate of TKA, and precise assessment of lower extremity alignment and component location is thought to be the primary determinant of a good clinical outcome over the long term. Attaining neutral post-operative limb alignment (within 3° of varus/valgus relative to the mechanical axis) has been one long-term goal of TKA [21]. Drawing a line from the femoral head's center to the ankle joint's center yields the mechanical axis of the lower extremity, which corresponds to a 5°-7° valgus angle in relation to the tibiofemoral anatomic alignment. By cutting the proximal tibia and distal femur perpendicular to the mechanical axis, neutral alignment could be achieved [22,23].

Functional analysis was finished using the KSS [24,25]. The mean KSS and mean function scores from a previous prospective study were reported to be 86.7 and 85.0, respectively. In our investigation, which covered 47 knees, alignment within 3 degrees of the neutral axis was seen, indicating a significant improvement in post-operative mechanical axis. Moreover, 56% of those patients obtained a satisfactory KSS, which was consistent with results from past studies. The results of our radiological investigation clearly demonstrated that in accordance with the KSS, an axis deviation of fewer than three degrees from neutral is associated with a high percentage of favorable clinical and functional outcomes [26].

Like in most recent investigations, the present study's permissible range of alignment led to a decreased revision rate. This means that in order to keep the prosthesis from failing, post-operative neutral alignment restoration may be necessary. This research has several restrictions. Secondly, a more specific definition of the optimal target value for alignment following TKA is not possible. In addition, more patients are required to support our case and justification for neutral mechanical alignment. Another drawback is the outlier group's tiny size in comparison to the acceptable group.

## Conclusions

A better knee flexion and knee score can be reached in patients with optimal component placement and precise rotational alignment. We arrive at the conclusion that the proper axial and rotational alignment of the components enhances the functional outcome of the fixed-bearing knee in low-demand patients with an excellent long-term clinical outcome. Lower revision rates and greater durability after total knee arthroplasty (TKA) are made possible by a mechanical axis that is properly oriented. Lower revision rates after TKA and increased longevity may be caused by the restoration of neutral limb alignment. To obtain a firm conclusion, additional testing on a larger sample of patients is required.
